# OECD indicator ‘AMI 30-day mortality’ is neither comparable between countries nor suitable as indicator for quality of acute care

**DOI:** 10.1007/s00392-023-02296-z

**Published:** 2023-09-08

**Authors:** Susanne Stolpe, Bernd Kowall, Karl Werdan, Uwe Zeymer, Kurt Bestehorn, Michael A. Weber, Steffen Schneider, Andreas Stang

**Affiliations:** 1 Institute for Medical Informatics, Biometry and Epidemiology (IMIBE), University Hospital Essen, Hufelandstr 55, 45147 Essen, Germany; 2 Center for Health Services Research of the German Cardiac Society, Düsseldorf, Germany; 3 Department of Medicine III, University Hospital Halle (Saale), Martin-Luther-University Halle-Wittenberg, Halle, Germany; 4 Foundation IHF, Institute for Myocardial Infarction Research, Hospital Ludwigshafen, Ludwigshafen, Germany; 5 German Society for Prevention and Rehabilitation of Cardiovascular Diseases e.V., Koblenz, Germany; 6 Institute for Clinical Pharmacology, Technical University Dresden, Dresden, Germany; 7 Association of Senior Hospital Physicians in Germany e.V., Düsseldorf, Germany; 8 Department of Epidemiology, School of Public Health, Boston, MA USA

**Keywords:** AMI 30-day-mortality, AMI hospital mortality, Quality of acute care, OECD indicator of quality of acute care, Trends in AMI hospital mortality

## Abstract

**Background:**

Hospital mortality after acute myocardial infarction (AMI, ICD-10: I21–I22) is used as OECD indicator of the quality of acute care. The reported AMI hospital mortality in Germany is more than twice as high as in the Netherlands or Scandinavia. Yet, in Europe, Germany ranks high in health spending and availability of cardiac procedures. We provide insights into this contradictory situation.

**Methods:**

Information was collected on possible factors causing the reported differences in AMI mortality such as prevalence of risk factors or comorbidities, guideline conform treatment, patient registration, and health system structures of European countries. International experts were interviewed. Data on OECD indicators ‘AMI 30-day mortality using unlinked data’ and ‘average length of stay after AMI’ were used to describe the association between these variables graphically and by linear regression.

**Results:**

Differences in prevalence of risk factors or comorbidities or in guideline conform acute care account only to a smaller extent for the reported differences in AMI hospital mortality. It is influenced mainly by patient registration rules and organization of health care. Non-reporting of day cases as patients and centralization of AMI care—with more frequent inter-hospital patient transfers—artificially lead to lower calculated hospital mortality. Frequency of patient transfers and national reimbursement policies affect the average length of stay in hospital which is strongly associated with AMI hospital mortality (adj *R*^2^ = 0.56). AMI mortality reported from registries is distorted by different underlying populations.

**Conclusion:**

Most of the variation in AMI hospital mortality is explained by differences in patient registration and organization of care instead of differences in quality of care, which hinders cross-country comparisons of AMI mortality. Europe-wide sentinel regions with comparable registries are necessary to compare (acute) care after myocardial infarction.

**Graphical abstract:**

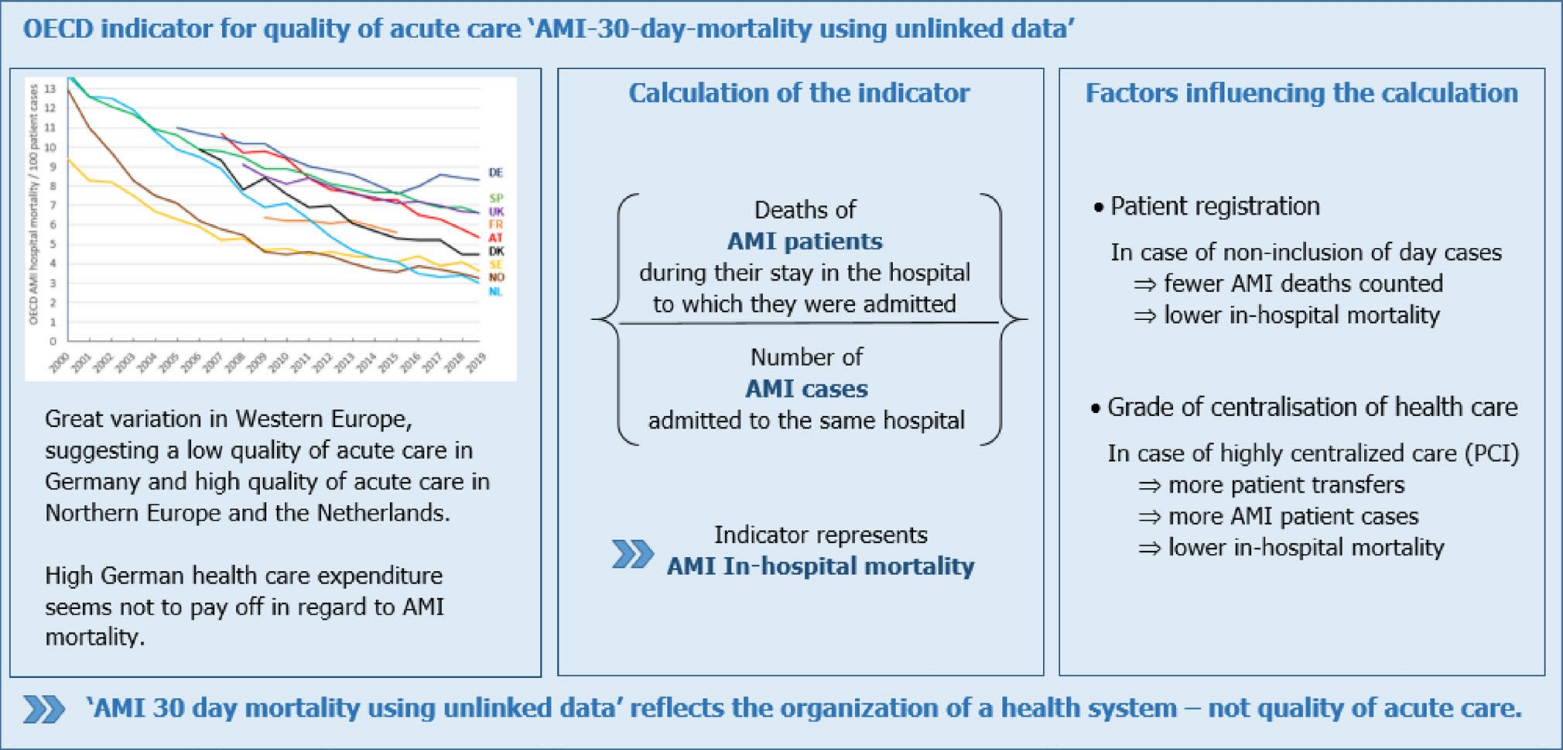

**Supplementary Information:**

The online version contains supplementary material available at 10.1007/s00392-023-02296-z.

## Background

The OECD (Organization for Economic Cooperation and Development) uses patient outcome after acute myocardial infarction (AMI, ICD-10: I21, I22) as indicator for assessing the quality of acute care in a country. An analysis of German health experts on quality of care in Germany showed that—in spite of the highest number of percutaneous transluminal coronary angioplasties in Europe—the German ‘AMI 30-day mortality’ is among the highest in Europe. Compared to other European countries reporting much fewer procedures but markedly lower AMI 30-day mortality, it was concluded that in Germany, health care overuse does not save lives [1].

**Fig.1:**
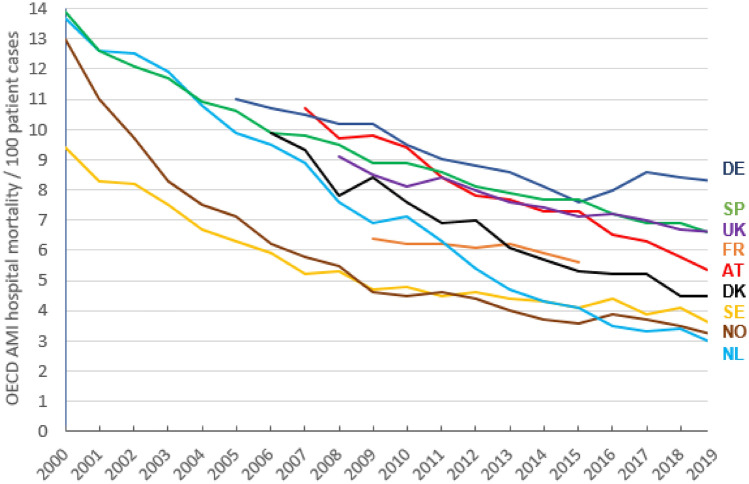
OECD AMI hospital mortality (indicator: AMI 30-day-mortality using unlinked data) from 2000 to 2019 for selected European countries. *AT*   Austria, *DE*  Germany, *DK*  Denmark, *FR*   France, *NL*  The Netherlands, *NO*   Norway, *SE*  Sweden, *SP*  Spain, *UK* United Kingdom Source: https://stats.oecd.org

According to OECD, AMI hospital mortality (‘AMI 30-day mortality using *unlinked* data’) has decreased in European countries since 2000, but from different levels and in different ways (Fig. 1). In Norway and Sweden, AMI mortality showed a steep decrease already until 2009 and declined further continuously—but at lower pace. In Spain and the Netherlands, AMI mortality declined uniformly until 2006 to about 10%. Then, the decline in the Netherlands gained even greater momentum, leading to the lowest AMI hospital mortality in Europe in 2019. For Austria and Germany, OECD reported an AMI hospital mortality of about 11% in 2007. While it dropped continuously in Austria to 5.2% in 2019, it even increased in Germany after 2014. In 2019, OECD-reported AMI hospital mortality in Germany was 8.5%, compared to 3.2% in Norway and 2.9% in the Netherlands.

To understand and interpret these data correctly, it is necessary to take a close look at the definition of two variables used by the OECD to report on AMI mortality: ‘AMI 30-day mortality using *unlinked* data’ and ‘AMI 30-day mortality using *linked* data’ [2]. For Germany, only information for ‘AMI 30-day mortality using *unlinked* data’ is available. ‘*Unlinked* data’ means that information on outcome after AMI is only available for the time an AMI patient stayed in a hospital after admission. This indicator variable consequently represents the hospital mortality (or case fatality rates) of AMI within 30 days after hospital admission where the death occurred in the same hospital as the initial admission. This means that patient follow-up on average covers a considerably shorter time period than 30 days—contrasting to the indicator’s name.

To be consistent with the terms used in data sources and literature, we will use ‘AMI hospital mortality’ and ‘AMI mortality’ throughout this article as preferred terms instead of ‘AMI case fatality’, which would be used preferably in epidemiology in this context.

For the OECD indicator ‘AMI 30-day mortality using *linked* data’, AMI mortality is calculated using information on the vital status of a patient 30 days after the first admission and registration as AMI patient in a hospital. To achieve this, vital status information must be linked to a patient’s hospital data. However, in countries with strict data security regulations, such as Germany, it is prohibited to link patient data from different sources. Therefore, 30-day mortality after hospital admission for AMI cannot be routinely reported using hospital registries when patients are transferred between hospitals or discharged before 30 days. For these countries, AMI 30-day mortality can only be estimated using AMI registries, cohort studies or analyzing secondary data from health insurance companies [6–8].

Interpreting the OCED variable ‘30-day AMI mortality using *unlinked* data’ as indicator for quality of acute care, Germany seems to trail a great deal behind most European countries, in which efficient improvements in patient care seem to have been implemented. As Germany is among the top European countries in terms of health care expenditure, availability of cardiologists and number of interventional procedures [3], the high AMI hospital mortality is unexpected. This is also mentioned in an analysis in the Health System Review for Germany published by the European Observatory on Healthy System Policies referring to the OECD indicator “30-day AMI mortality” [3]. The authors conclude that there is room for improvement in quality of in-patient health care in Germany, especially for hospital mortality after AMI and that—given the high health expenditure—some health outcomes are only moderate.

Interestingly, other OECD indicators for quality of acute care such as ‘30-day mortality after admission to hospital for ischemic stroke based on *unlinked* data’ show a different picture. In 2019, ischemic stroke hospital mortality in Germany was average among European countries, (OECD library: Health at a Glance 2021: OECD indicators, 10.1787/ae3016b9-en). If the OECD indicator ‘AMI 30-day mortality using *unlinked* data’ reflected quality of acute care, the comparable low quality of care in Germany should negatively affect hospital mortality after admission for ischemic stroke as well. Yet, this seems not to be the case. However, reasons for these differences in the OECD quality indicators have not discussed before [3].

A closer look at how the indicator ‘AMI 30-day mortality using *unlinked* data’ is calculated shows that the numerator in the equation is patient based, while the denominator is based on patient cases:

$$\normalsize {\text{AMI 30}}{\text{ - day mortality using unlinked data}} = \frac{{{\text{Number of deaths among AMI patients}}\;{\text{during their stay in a hospital}}}}{{{\text{Number of cases}}\;{\text{that have been admitted to a hospital due to AMI }}}}.$$

As a consequence, every new hospital admission of a patient generates a new case. Therefore, one patient can correspond to more than one case if a patient was transferred between hospitals during AMI treatment.

The numerator in the equation includes all AMI patients that have been admitted to a hospital and died there. Here, all deaths of admitted AMI patients are counted, independent of the actual cause of death, which would be eventually mentioned on the respective death certificate. Therefore, AMI hospital mortality is not affected by differences in the quality of death certification—in contrast to the national AMI mortality rates [4].

This article addresses the questions, whether AMI hospital mortality (represented by the OECD indicator ‘AMI 30-day mortality using *unlinked* data’) really reflects quality of acute care in a health system and second, whether the quality of acute care can be inferred to and compared between countries using other data sources.

## Methods

Literature and public health reports on topics that might help to explain AMI hospital mortality were searched. Topics of interest included characteristics of AMI patient populations such as age, proportion of STEMI patients, risk factors and (co)morbidities (e.g., heart failure, renal failure, diabetes, hypertension), frequency of complications (such as cardiogenic shock), frequency of guideline conform treatment such as timely PCI and medication, and (pre-hospital) emergency service as well as time between onset of symptoms and treatment.

Additionally, we tried to find information on aspects of health service organization such as patient registration rules, degree of centralization of PCI facilities, and frequency of patient transfers.

We focused our project on those European countries for which we were able to understand information in documents that were only available in the national language: France, the Netherlands, the United Kingdom, Denmark, Norway, Sweden, Spain, Austria, and Germany.

International experts and authors of relevant articles (see acknowledgment) were contacted and interviewed on aspects regarding the organization of health care and patient documentation that could not be clarified using published literature alone.

OECD meta-data (https://qdd.oecd.org/subject.aspx?Subject=hcqo_meta; accessed 9.3.2022), documenting the features of the underlying national data sources, were reviewed.

The association between ‘AMI 30-day mortality using *unlinked* data’ and ‘Average length of stay after AMI’ was described graphically. The explained variation in hospital mortality (adjusted *R*^2^) was assessed by linear regression using SAS software Version 9.4, SAS Institute Inc. Cary, NC, USA.

## Results

### Patient characteristics and hospital care as potential factors to explain AMI hospital mortality

#### Differences in patient characteristics and acute care

Although there are numerous studies on outcome after AMI, a comparison of patient characteristics and acute care was not validly possible because of different included patient populations, different reported time periods and variables [5–11]. Comparisons between countries yielded inconsistent results regarding patient and treatment characteristics. For 2014–2017, a study comparing the treatment and outcome after STEMI in Sweden and Norway, Swedish patients were older, had more often hypertension (50% vs. 40%) and heart failure (9% vs. 3%) but smoked less (25% vs. 38%) [7]. Compared to the UK, comorbidities were more frequent in Sweden [12]. However, prevalence of classical risk factors for myocardial infarction seems to be not associated with a higher mortality risk [13].

Data from registries in Sweden, Norway, Germany, the UK, and France report different frequencies of PCI in STEMI patients [7, 12, 14–17]. However, these differences could not explain the differences in AMI hospital mortality. According to German registries, PCIs were done in about 80% of STEMI cases. A higher frequency was only reported in the French FAST-MI registry (90%). In Norway, having the second lowest AMI hospital mortality, only 66% of STEMI patients underwent a PCI [7].

#### Differences in emergency care for AMI patients

The routine use of telemedicine for pre-hospital AMI diagnosis in Denmark influences symptom-to-treatment time and mortality [18]. Compared to Germany [19], in Denmark, and UK, a greater proportion of patients with symptoms of myocardial infarction is admitted to hospital via the ambulance service reducing pre-hospital times [20].

Out-of-hospital cardiac arrests (OHCA) can be attributed to cardiac causes in about 45%–70% [21, 22]. The incidence of OHCA in 2017 was higher in Germany (107/100,000) than in Denmark (86/100,000) and the Netherlands (59/100,000) [23]. Early resuscitation increases survival rate. In 2017, the rate of bystander cardiopulmonary resuscitation (CPR) was higher in Norway (in 83% of all OHCA cases) than in Denmark (70%) and in Germany (46%) [21]. Use of defibrillators is particularly widespread in the Netherlands (23–59% of all OHCA cases), in contrast to Sweden (15%) and Denmark (4%) [21]. Survival among all OHCA patients (medical and non-medical causes) was 9–13% in Germany, 17% in Denmark, and 23% in the Netherlands [22, 23].

### Organization of health care systems influencing AMI hospital mortality

#### Reporting of day cases in OECD data sources

Patients admitted to hospital for only a short time are categorized as “hourly cases” or “day cases”. In Europe, different definitions for “day case” or “hourly case” exist: Patients whose admission and discharge dates are the same (Germany), patients who were in the hospital for less than 24 h (Denmark, Sweden), patients who did not spend a day of care or a night in the hospital (the Netherlands), or patients who spent less than 24 h in the hospital or who did not receive a procedure during their hospital stay (France).

Mortality risk after AMI is particularly high in the first hours after onset. If day or hourly cases are not registered as hospitalized, deaths after AMI within less than 24 h are not reported and counted. Consequently, calculated AMI hospital mortality will be lower.

In the OECD meta-data (see Supplementary Table S1), all selected countries report that day cases are included in the national data source for calculating the indicators on AMI mortality. However, examining the patient registration regulations in the selected countries, this seems questionable as illustrated below.

#### Inclusion of day cases in the Dutch data source

The Netherlands report the lowest AMI hospital mortality in Europe (2.9% in 2019). According to the report of the Dutch Heart Society 2019, the patient registration regulations in the Netherlands changed in 2012 [24]. Since then, a patient is registered as in-patient, if one day of care and one overnight stay in hospital is registered. Since 2014, the category ‘observation’ (in the original: “observatie”) was introduced for patients without an overnight stay. Unscheduled admissions, previously treated as 1-day admissions, have since been “probably” (in the original: “waarschijnlijk”) registered primarily as ‘observation’. OECD-reported AMI hospital mortality declined at greater pace after these changes.

In a mortality forecast of the Dutch Heart Society, AMI patients registered as ‘observations’ were explicitly included to predict the 30-day AMI mortality for 2018 in and out of hospital ([11], Table 2.1). The forecast estimated a 30-day AMI mortality of 10.6% after first hospital admission. Yet, OECD reported a 30-day AMI mortality in and out of hospital of only 4%. This seems to indicate that day cases appear not to be included in the Dutch OECD data source.

#### Inclusion of day cases in the French data source

In 2015, the last reported the OECD AMI hospital mortality for France was 5.8%. The data source provided to OECD to calculate hospital mortality then was the ‘Programme de Médicalisation des systèmes d’information’ registry (Supplementary Table S1). It comprises patient admissions, procedures, and patient care information. Hourly cases are not included in this database [25]. The ‘Résumé de passage aux urgences’ which documents emergency cases is neither mentioned as source in the OECD meta-data.

#### Inclusion of day cases in data sources from Norway, Sweden, and Denmark

Patients with one overnight stay or a minimum number of hours in hospital are considered as hospitalized. We could not find any documentation on this in English language and relied on statements from experts (see acknowledgement). It is not clear if day cases are included in the OECD source data for Sweden and Denmark. For Norway, an analysis of the Patient Administrative System, comprising patient data from all Norwegian hospitals, reported an AMI hospital mortality of 7.8% for 2016 [26]. This figure is considerably higher than the reported mortality by the OECD (3.9%) based on the Norwegian Patient Registry (Supplementary Fig. S2).

#### Day cases reported by the United Kingdom and Austria

Health care systems in the United Kingdom (UK) and Austria do not distinguish day cases from in-patients. For Austria, AMI mortality in 2017 according to the Austrian Hospital Quality Report was 5.6% [27] and even lower than in the OECD report (6.3%) (Supplementary Fig. S2).

#### Reporting of day cases in Germany

In Germany, day cases are documented separately, but included in the national OECD data source. AMI hospital mortality using publicly available information on all hospital patients including day cases was 8.2% for 2018 (gbe-bund.de access on 20.5.2022). The small difference compared to the OECD-reported AMI hospital mortality can be caused by the use of different standard populations. OECD data on AMI mortality are standardized by the OECD disease population, which places a higher weight on older age groups compared to the German standard population 2011.

### Average length of stay in hospital after AMI

According to OECD, average length of hospital stay (LOS) after AMI in 2019 in Europe ranged from 3.4 days in Norway to 9.9 days in Germany (average: 6.5 days) (https://stats.oecd.org/). The longer a patient stays in hospital after AMI, the higher is the probability of death during that stay. If a patient stays 3 days in hospital, a potential death can occur lately on day 3 after onset. A patient staying 10 days in hospital—and is, therefore, alive at day 3—probably dies on the 10th day and is counted as AMI death in hospital.

**Fig. 2:**
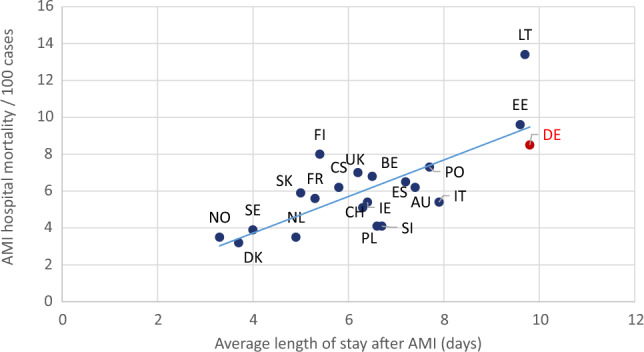
Association between AMI hospital mortality and average length of stay in hospital after AMI (days) in 2018 according to OECD. *AU* Austria, *BE* Belgium, *CH* Switzerland, *CS* Czechoslovakia, *DE* Germany, *DK* Denmark, *EE* Estonia, *ES* Spain, *FI* Finland, *FR* France, *IE* Ireland, *IT* Italy, *LT* Lithuania, *NL* the Netherlands, *NO* Norway, *PL* Poland, *PO* Portugal, *SE* Sweden, *SI* Slovenia, *SK* Slovakia, *UK* United Kingdom

Figure 2 shows the association between AMI hospital mortality and average LOS after AMI in 2018. The adjusted *R*^2^ is 0.56 and indicates, that almost 60% of the variation in the AMI hospital mortality is explained by the average LOS.

### Frequency of inter-hospital transfers of AMI patients

**Fig. 3:**
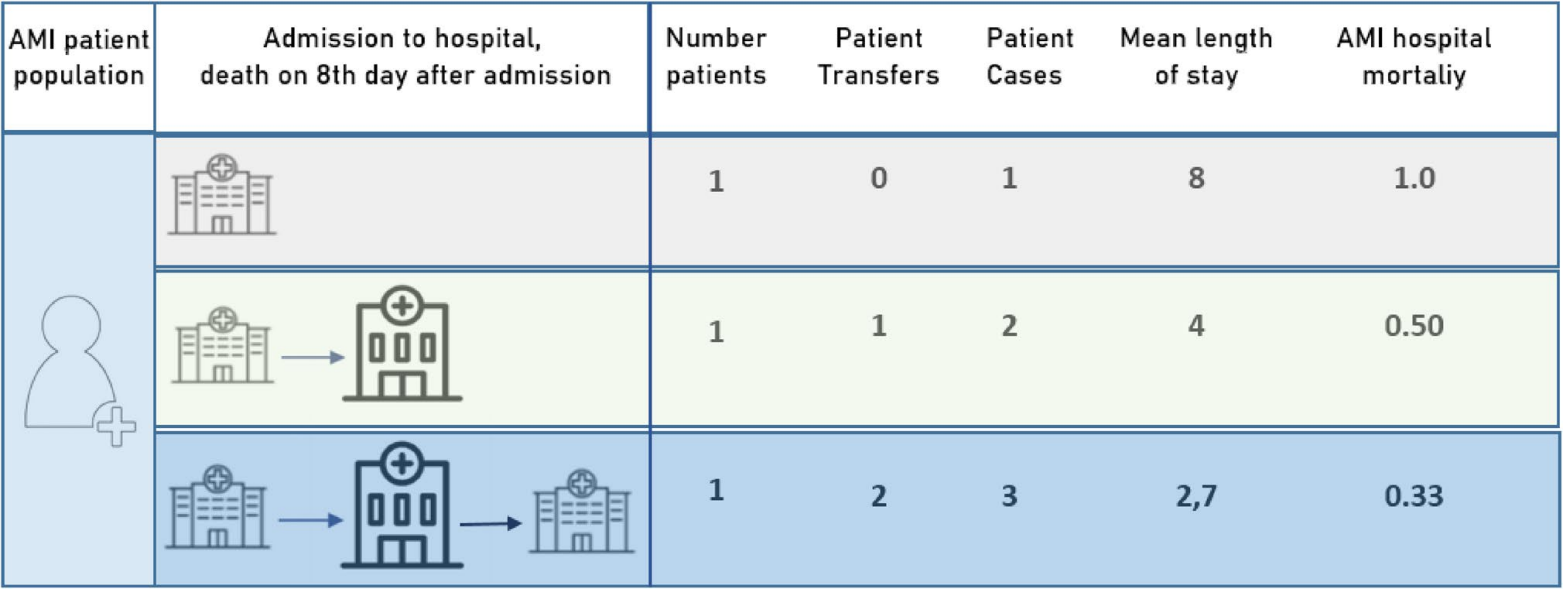
Potential courses of treatment after AMI—regarding inter-hospital transfers and the effect on calculated average length of stay and hospital mortality based on a population of one AMI patient

In countries with centralized PCI capacities such as Denmark, Norway, or Sweden, patients are regularly transferred from local hospitals to specialized hospitals offering PCI. After catheterization, patients are often re-transferred to the referring local hospital. A high grade of centralization leads to a high frequency of patient transfers—and eventually to a lower calculated hospital mortality: Having an equal number AMI deaths, these deaths (patient based) are related to a larger number of admissions (= cases) in the denominator when calculating hospital mortality. More frequent patient transfers reduce the average LOS and thereby, AMI hospital mortality (Fig. 3).

Transfer frequency of AMI patients was reported in German and Austrian hospital quality reports [27, 28]. Few studies reported the outcome of AMI after inter-hospital transfers of patients in defined areas [29–31]. By personal contact, we obtained information on frequency of patient transfers in France, Norway, and Denmark (Table 1). Frequency of patient transfers in cases of AMI seem to range from 17% in Germany to 50% and more in Norway and Denmark.

**Table 1:** Transfer frequencies of AMI patients in Europe

Country	Percentage of AMI patients with inter-hospital transfers	Sources
Germany	17% (2018)	German Inpatient Quality Indicators (G-IQI) 2018 [28]
The Netherlands	21% (2008–2010)	Study with 846 patients of the University Medical Center Groningen [32]—potential retransfers were not reported
Austria	24% (2018)	Austrian Inpatient Quality Indicators (A-IQI) 2018 [27]
Switzerland	28% all AMI, 49% in patients with PCI (2019)	Calculated from quality indicators of Swiss Acute Care Hospitals 2019 [33]
Sweden	27% (2020)	Evaluation from the Svensk Patientregistret (done by Jonathan Lindström, Dept. of registration and statistics, Swedish Patient Registry, Stockholm)
United Kingdom*	26% (2005–2015), London area	Cohort Study, London [29]
France	29% (2010–2014, 2015)	Report from the RESURCOR networks, French Alps, 2010–2014 [30], evaluation from FAST-MI 2015 (by Prof. Nicolas Danchin, Principal Investigator FAST-MI);
Denmark	72% (1997–2001)	DANAMI-2- multicenter Study [31]
Norway	50% of AMI patients are treated in more than one hospital (2021)	Information by Prof. Jon Helgeland, head of the institute for public health, Oslo (now retired)

*According to OECD meta-data on indicators for quality of acute care, admissions which result in a transfer to another acute care hospital are not included in the calculation of admission based indicators in UK (see Supplementary Table S1). Therefore, AMI hospital mortality in UK might not be as strongly influenced by a higher transfer frequency as in other countries

Using this information on frequency of patient transfers, it can be calculated how many patients account for 100 patient cases (= admissions). Assuming, a patient is transferred only once, with a transfer frequency of 17% in Germany, 85 patients create 100 patient cases (Supplementary Table S3).

In Norway, with 50% of patients transferred, 67 patients create 100 cases. If it assumed that 50% of the transferred patients are re-transferred after PCI, 80 patients create 100 cases in Germany, compared to only 57 patients in Norway. If hospital mortality is recalculated based on AMI patients instead of patient cases the influence of different transfer frequency can be eliminated (Supplementary Table S3). After the patient-based recalculation of AMI hospital mortality, differences between countries decrease, but they do not disappear.

### AMI hospital mortality as reported from registries

AMI registries might be more suitable to report patient outcome and to assess quality of acute care. Outcome after AMI is reported from registries in France (FAST-MI), the United Kingdom (MINAP), Sweden (SWEDEHEART), Austria (VIENNA-STEMI), and Germany (Augsburg Myocardial Infarction Registry, KORA) (Supplementary Table S4). However, it is difficult to retrieve actual and comparable information on outcome after AMI during a stay in hospital [12, 14, 16, 26–28, 34, 35]. Compared to the OECD-reported data, AMI hospital mortality reported from registries was comparable for Germany, Austria, Sweden, and the UK, but differed strongly for Norway and France (Supplementary Fig. S2).

## Discussion

OECD-reported AMI hospital mortality (‘AMI 30-day mortality using *unlinked* data’) is not suitable to validly reflect quality of acute care in a health system. Differences in the indicator are mainly caused by differences in patient registration and organization of national health systems.

### AMI hospital mortality as indicator of quality of care

Selecting a variable as indicator for quality of acute care is based on the assumption that this variable will reflect level and changes of quality of acute care. The course of AMI hospital mortality as shown in Fig. 1 illustrates that since 2000, the quality of acute care improved in Europe continuously over time as AMI hospital mortality declined, which is supported by progress in AMI diagnostics and treatment during the last 20 years.

However, as it comes to direct comparisons between countries, the obvious conclusions from Fig. 1, that the quality of acute patient care after AMI is quite divers in Europe and is much better in the Netherlands or Sweden than in Germany or Austria, seems questionable.

Additionally, the steep declines in AMI hospital mortality shown in Fig. 1 do not indicate the introduction of even more effective improvements in acute patient care (such as in in Norway in 2000, in Denmark in 2009 or in the Netherlands in 2010), but must first be interpreted as points in time with changes in elements of health care organization.

In Europe, it can be expected that results from AMI research spread quickly. This especially in the high-income countries included in our comparison that provide universal health care of comparable quality to their citizens. Another OECD indicator for acute care, the variable “hospital mortality after ischemic stroke” confirms this—showing a comparable stroke mortality for all selected countries.

Differences and changes in baseline characteristics of AMI patients regarding the “classical” factors that affect AMI hospital mortality—relating to patient age, AMI severity, prevalence of risk factors such as smoking or overweight, and comorbidities such as diabetes, heart failure or renal disease—seem to have only minor influence. In fact, AMI patients in the selected countries differ in their baseline characteristics—but in varying ways. Nowhere was the prevalence of all relevant risk factors unfavorably high or favorably low. Therefore, none of these factors can have as great an influence on AMI hospital mortality as necessary to explain the differences between the respective countries and especially to explain the strikingly high AMI mortality in Germany. Indeed, it has recently been shown that the prevalence of known risk factors for myocardial infarction does not seem to be associated with a higher mortality risk [36].

Differences in the organization of emergency care [37] and in the rate of bystander CPR in cases of OHCA could influence AMI hospital mortality. However, although patients receiving early CPR have a greater chance to reach a hospital alive, their mortality risk after admission is very high [38]. OHCA patients with less optimal early emergency care might die before reaching the hospital and would not be considered when calculating AMI hospital mortality. It is, therefore, not possible to assess whether differences in pre-hospital care of OHCA can explain part of the differences in AMI hospital mortality.

The provision of guideline conform AMI therapy—namely timely PCI—is not only driven by the recognized necessity in case of STEMI, but is postulated also to be driven by existing reimbursement regulations [39]. In Germany, since 2020, the German Institute for Medical Documentation and Information (DIMDI) annually received a request for introducing a new OPS code for reimbursement of emergency PCIs, because costs of PCI care outside regular working hours-especially at night-are not covered to full extent [40]. The impact on AMI hospital mortality of possible underfunding of out-of-hour PCI, which could lead to delayed PCI in patients that were admitted at night or at weekends, cannot be estimated.

Unexpectedly, factors relating to health system organization have a large impact on the calculation of hospital mortality. We identified differences in patient registration, frequency of patient transfers and length of stay in hospital as major influencing factors.

### Patient registration

Patient registration rules regarding the documentation of day cases affect mortality calculation especially for diseases with a high fatality during the first day. However, information on patient registration in a country was scarce. If any, it was provided mostly in a country’s own language. This is astonishing, as this information is important to assess the completeness and comparability of patient reporting in the national data sources.

Mortality risk after admission is highest during the first 24 h. Simon et al. reported an AMI fatality between 2% (for men, 30–67 years) and 10% (for women, 68–89 years) for the first day after admission [41]; Malacrida et al. reported a mortality risk of 4.3% for men and 7.1% for women [42]. Taking the reported AMI mortality risk during the first hours as example, countries that do not document day cases in the source data provided to the OECD might miss about 50% of all in-hospital deaths after AMI [42] and about 30% (for younger patients) and 50% (of older patients) of all AMI deaths within 30 days [41]. In case of the Netherlands, it seems necessary to correct the reported AMI hospital mortality by these factors.

### Average length of stay in hospital

Average LOS is strongly associated with AMI hospital mortality. LOS after AMI is influenced by disease severity, frequency of severe comorbidities [43] or complications during hospital stay which by themselves affect AMI mortality. Unfortunately, reports that allow valid comparisons of these variables are rare. However, LOS is affected by other aspects as well: hospital reimbursement policies affect the time of patient discharge to maximize profit and, frequent inter-hospital patient transfers lead to shorter LOS in a hospital—which itself is affected by reimbursement policies as well.

Especially, a larger frequency of patient transfers affects AMI hospital mortality. Therefore, AMI hospital mortality cannot be compared validly without data on transfer frequency in AMI patients. Information on the frequency of patient transfers is rare and not easy to find. It can be assumed that in general, the transfer frequency is lower in countries where many hospitals are able to provide PCIs. In order to compare AMI hospital mortality, information about all the organizational background of patient care are needed.

## AMI 30-day mortality, a more valid indicator?

Patient-related 30-day in and out of hospital mortality is more appropriate for comparing the quality of acute care after AMI [44]. AMI 30-day mortality is not affected by the frequency of patient transfers or LOS. However, it is affected as well by the specifics of patient registration. Unfortunately, due to data security regulations, this indicator is not universally available for international comparisons. AMI 30-day mortality reported from registries is equally divers due to differences in included populations and applied definitions of variables and does not allow for valid comparisons.

Valid comparisons of AMI hospital mortality, AMI 30-day mortality and quality of acute care in AMI seem only to be feasible using registries that apply the same inclusion and exclusion criteria for their patients and hospitals and that are situated in regions with comparable population structure (sentinel regions), to secure representativeness.

## Strengths and limitations

We focused on France, the Netherlands, Great Britain, Denmark, Norway, Sweden, Spain, Austria, and Germany, as health reports from these countries could be either translated or were available in English. However, we are rather confident, that we did not miss important aspects that might add substantially to the explanation of differences in AMI hospital mortality between European countries.

Information on organization of health care was only scarce, especially on rules of patient registration or frequency of patient transfers. For some countries, we had to rely on expert information only.

## Conclusion

Reliable international comparisons of quality of acute care using AMI hospital mortality as indicator are only possible to a limited extent. AMI hospital mortality reflects structural differences between health systems regarding patient registration, centralization of PCI facilities and hospital reimbursement policies. Patient outcome after AMI reported from registries is influenced by the selected patient population, which equally hinders valid comparisons. Valid comparisons are only possible using methodologically comparable registries. A European-wide monitoring of AMI mortality based on representative sentinel regions with uniform reporting and inclusion criteria is missing so far and could contribute to a valid comparison of the quality of care.

## Supplementary Information

Below is the link to the electronic supplementary material.

Supplementary file1 (DOCX 27 KB)

## Data Availability

Data used for this project are publicly available via the OCED website https://stats.oecd.org.
