# Correction: OECD indicator ‘AMI 30-day mortality’ is neither comparable between countries nor suitable as indicator for quality of acute care

**DOI:** 10.1007/s00392-023-02342-w

**Published:** 2023-11-22

**Authors:** Susanne Stolpe, Bernd Kowall, Karl Werdan, Uwe Zeymer, Kurt Bestehorn, Michael A. Weber, Steffen Schneider, Andreas Stang

**Affiliations:** 1 Institute for Medical Informatics, Biometry and Epidemiology (IMIBE), University Hospital Essen, Hufelandstr 55, 45147 Essen, Germany; 2 Center for Health Services Research of the German Cardiac Society, Düsseldorf, Germany; 3 Department of Medicine III, University Hospital Halle (Saale), Martin-Luther-University Halle-Wittenberg, Halle, Germany; 4 Foundation IHF, Institute for Myocardial Infarction Research, Hospital Ludwigshafen, Ludwigshafen, Germany; 5 German Society for Prevention and Rehabilitation of Cardiovascular Diseases e.V., Koblenz, Germany; 6 Institute for Clinical Pharmacology, Technical University Dresden, Dresden, Germany; 7 Association of Senior Hospital Physicians in Germany e.V., Düsseldorf, Germany; 8 Department of Epidemiology, School of Public Health, Boston, MA USA

**Correction: Clinical Research in Cardiology** 10.1007/s00392-023-02296-z

During the proof-process, a reference had been accidentally removed. In paragraph 3 of the ‘Background’-section (at the end of the first sentence), the following reference should have been added:

“Heber R, Levsen A, Offermanns M. Meaningfulness of hospital structure and quality comparisons based on OECD data. [Aussagekraft von Krankenhausstruktur- und Qualitätsvergleichen auf Basis von OECD-Daten. Düsseldorf: Deutsches Krankenhausinstitut; 2021”.

The Original Article has been updated.

