# Supplementary material for: OECD indicator ‘AMI 30-day mortality’ is neither comparable between countries nor suitable as indicator for quality of acute care
Source: Clin Res Cardiol. 2023 Sep 8;113(12):1650–60. doi: 10.1007/s00392-023-02296-z (PMC11579078; doi:10.1007/s00392-023-02296-z)
Supplement: Supplementary file 1 — Supplementary file1 (DOCX 27 KB) [file 392_2023_2296_MOESM1_ESM.docx]

**Content of supplementary file**

Table S1:
OECD Meta-Data on data sources for reporting indicators of quality of acute care (‚AMI 30-day mortality using unlinked data‘). Selected information and countries with reporting patient data 2016/2017 to OECD.
Source: https://stats.oecd.org/index.aspx?queryid=51881). HCQO-Meta Data, OECD (<https://qdd.oecd.org/subject.aspx?Subject=hcqo_meta>)

Figure S2:
AMI hospital mortality – as reported by OECD (dark bars) compared with the respective figures from other national data sources or registries (light bars).

Table S3:
Frequency of inter-hospital transfers of AMI patients, number of patients that contribute to 100 cases using two different transfer scenarios, and recalculated patient-based hospital mortality based on the ‘real’ number of patients. Patient-based hospital mortality is calculated for three case based hospital mortalities (4%, 6% and 8%).

Table S4:
Selection of European Registries including AMI patients.

Supplementary Table S1. OECD Meta-Data on data sources for reporting indicators of quality of acute care (‚AMI 30-day mortality using unlinked data‘). Selected information and countries with reporting patient data 2016/2017 to OECD.
Source: Health Care Quality Incidators (https://stats.oecd.org/index.aspx?queryid=51881). HCQO-Meta Data, OECD (https://qdd.oecd.org/subject.aspx?Subject=hcqo_meta)

|  | Austria | Denmark | France | Germany | Netherlands | Norway | Spain | Sweden | UK |
| --- | --- | --- | --- | --- | --- | --- | --- | --- | --- |
| Intra-hospitale Mortalität 2018 | 5,8 | 4,5 | 5,6 (2015)* | 8,6 (2017) | 3,4 | 3,5 | 6,9 | 4,1 | 6,7 |
| Name of the Information System | Dokumentations- und Informationssystem für Analysen im Gesundheitswesen (DIAG) | National Patient Register | Programme de Médicalisation des systèmes d'information (PMSI) | DRG Statistics | LMR and its successor LBZ (National Medical Registry and National Basic Register Hospital Care) | Norwegian Patient Registry | National Hospital discharge database | Swedish Patient Register and Swedish Cause of Death Register | Patient Episode Database Wales/Admitted Patient Care |
| Completeness | Yes | Yes | Yes | Yes | No(not complete)  74% of the hospital admissions.  2018/19: complete | Yes | Yes | Yes | Yes |
| Are only acute non-elective (urgent) admissions included in the calculation of the indicators? | Yes | Yes | Yes | No (2018/19: Yes) | Yes | Yes |  | Yes | Yes |
| Base for the principal diagnosis (PDx) codes included in the dataset | Diagnosis demanding most resources | discharge diagnosis, based on main reason for admission | admission diagnosis,  main reason for admission | discharge diagnosis,  main reason for admission | discharge diagnosis - demanding most resources | admission diagnosis, demanding most resources | admission diagnosis,  main reason for admission | discharge diagnosis - demanding most resources | discharge diagnosis, main reason for admission |
| Were all relevant day cases included in the calculation of the numerator and denominator? | Yes | Yes | Yes | Yes | Yes | Yes | Yes*** | Yes | Yes |
| For admission-based indicators, were admissions which result in a transfer to another acute care hospital included in the calculation? | Yes | Yes** | Yes | Yes | Yes |  | Yes | Yes | No |

* last year of reported outcome after AMI
** „We added to the criteria that the contact has to be acute non-elective. In earlier data delivery, the admission-based indicators include all deaths. This has been corrected so that the numerator in the admission based Mortality-indicators only include deaths during admission. We have corrected our programming so that the year in the numerator is the year of admission and not the year of the deaths (there is now consistency between the years used in the denominator and the numerator).
*** „Same day cases are included both numerator and denominator but only if the patient have died in the hospital after being formally admited. Deaths in emergency room sevices have not been included.“

Supplementary Figure S2.
AMI hospital mortality – as reported by OECD (dark bars) compared with the respective figures from other national data sources or registries (light bars).

Sources: 9, 11, 13, 22, 23, 30, 31

Supplementary Table S3:
Frequency of inter-hospital transfers of AMI patients, number of patients that contribute to 100 cases using two different transfer scenarios, and recalculated patient-based hospital mortality based on the ‘real’ number of patients. Patient-based hospital mortality is calculated for three case based hospital mortalities (4%, 6% and 8%).

|  | Number of AMI patients that account for 100 AMI cases assuming the overall transfer frequency in a) and two different re-transfer-scenarios | | Patient based re-calculated hospital mortality*** (%) using number of patients accounting for 100 cases – under the assumption of different transfer frequencies  [b] [c] [b] [c] [b] [c] | | |
| --- | --- | --- | --- | --- | --- |
| a) Transfer- Frequency | b)  transferred patients (no re-transfer) | c)  transferred patients (50% of patients under b) are re-transferred after PCI) | Mortality  (case based)  8% | Mortality  (case based)  6% | Mortality (case based)  4% |
| 17% | 85* | 80** | 9,4 10,0 | 7,1 7,5 | 4,7 5,0 |
| 24% | 81 | 74 | 9,9 10,8 | 7,4 8,1 | 4,9 5,4 |
| 27% | 79 | 71 | 10,1 11,2 | 7,6 8,4 | 5,1 5,6 |
| 30% | 77 | 69 | 10,3 11,6 | 7,8 8,7 | 5,2 5,8 |
| 50% | 67 | 57 | 12,0 14,0 | 9,0 10,5 | 6,0 7,0 |

*Assumption: each transferred patient generates 2 patient cases. This means, that with a transfer frequency of 17% (as under a)) 17 of 100 patients generate 2 patient cases. Then, it needs 85 patients to generate 100 cases (result under b)).
**Assumption: 50% of all transferred patients (according to a)) are re-transferred after PCI. These re-transferred patients generate 3 cases. In case of a transfer-frequency of 17%, 8.5% of the AMI patients (17% x 50%= 8.5%) generate 3 patient cases. All other transferred AMI patients generate 2 cases. Under this assumption, 80 AMI patients generate 100 AMI cases.
*** Re-calculation of the AMI hospital mortality (changing case-based to patient-based denominator): in case of a hospital mortality of 8% (case-based, as reported by OECD), a patient based calculation – using the patient number under b) yields 9.4%; with the transfer scenario under c) yields a patient based hospital mortality of 10%.

Supplementary Table S4:
Selection of European Registries including AMI patients.

| Registry | Country | Inclusion criteria | Period |
| --- | --- | --- | --- |
| Myocardial infarction registry Augsburg* | DE | AMI; AMI mortality includes death from AMI or other coronary causes 25-84 years (since 2021: >25 years) | ongoing, Augsburg region |
| German myocardial infarction registry (DHR) | DE | STEMI-< 24h 243 voluntary hospitals | 09/2007-08/2008 |
| FITT-STEMI Registry | DE | STEMI < 24h Patients from PCI centers | Since 01/2009, ongoing- |
| SWEDEHEART | SE | ACS Patients with symptoms of AMI in ‚coronary care units‘ and ‚other specialized facilities‘ | ongoing, nationwide |
| NHR (Nederlands Hart Registratie) | NL | Patients with myocardial infarction and a cardiac procedure | ongoing, nationwide |
| MINAP (Myocardial Infarction National Audit Project) | UK | STEMI (Type 1)-Patients  Admission via emergency service (EMS), voluntary hospitals | Ongoing, nationwide |
| FAST-MI 2010 | FR | NSTEMI/STEMI < 48h Patients from cardiologic (intensiv-) care units; Participation of hospitals voluntarily (2015: 78%) | 10/2010 – 11/2010 |
| FAST-MI 2015 | FR |  | 10/2015 |
| SCALIM | FR | STEMI < 24 h | 2011-2014 |
| AMIS PLUS | CH | Acute myocardial infarction < 48 h at admisson or during hospital stay (STEMI/NSTEMI) Participation of hospitals voluntarily | ongoing, nationwide |
| VIENNA-STEMI (STEMI Registry Vienna) | AU | STEMI patients from 5 cardiologic centers (>400 PCI yearly) | ongoing, Vienna |

ACS: Acute coronary syndrome, AMI: acute myocardial infarction, AMIS Plus: Acute Myocardial Infarction in Switzerland, FAST-MI: French registry of acute STEMI or non-STEMI. FITT-STEMI: **F**eedback-**I**ntervention and **T**reatment-**T**imes in **ST**-**E**levation **M**yocardial **I**nfarction. NSTEMI: Non-ST-segment elevation myocardial infarction, SCALIM: Syndrome Coronaire Aigu en Limousin, STEMI: ST-segment elevation myocardial infarction. SWEDEHEART: Swedish Web System for Enhancement and Development of Evidence-Based Care in Heart Disease Evaluated According to Recommended Therapies.
